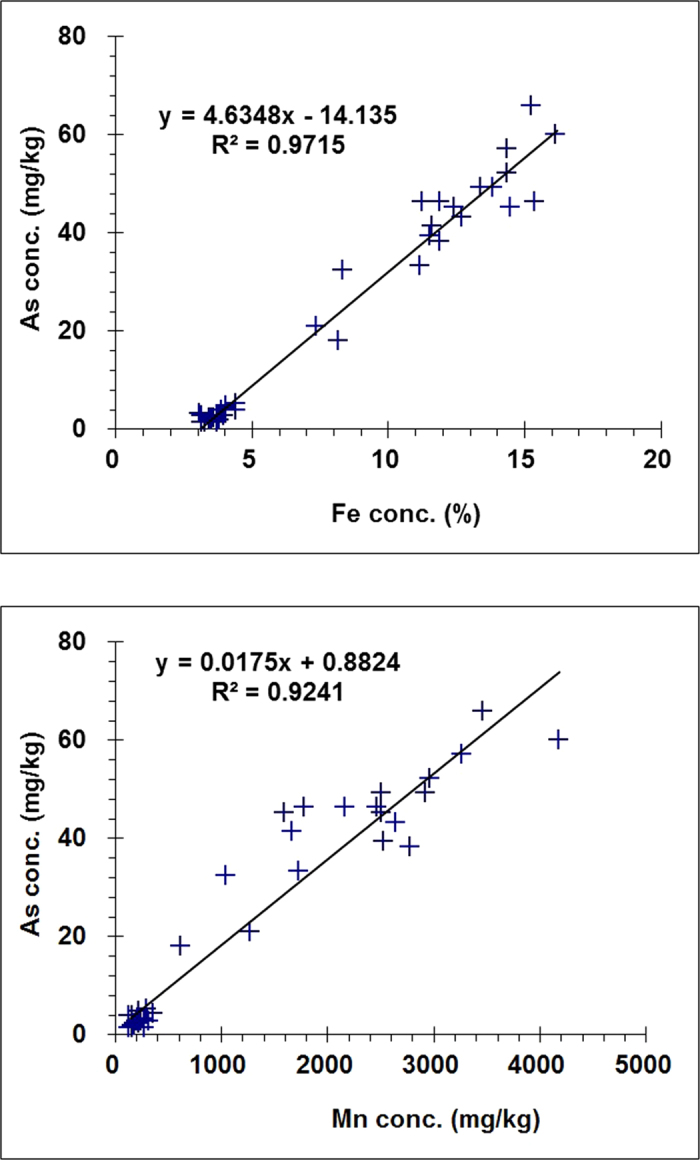# Arsenic profile distribution of the wetland argialbolls in the Sanjiang plain of northeastern China

**DOI:** 10.1038/srep10766

**Published:** 2015-06-04

**Authors:** Chunye Lin, Jing Wang, Hongguang Cheng, Wei Ouyang

**Affiliations:** 1 State Key Joint Laboratory of Environmental Simulation and Pollution Control, School of Environment, Beijing Normal University, Beijing, 100875, China; 2 China Land Surveying and Planning Institute, Ministry of Land and Resources, Beijing 100035, China

## Abstract

The wetland Argialbolls pedon was chosen to investigate the effects of pedogenic processes and anthropogenic activities on the vertical distribution of As concentrations. Two wetland Argialboll cores (90 cm long) were collected from the Sanjiang Plain in northeastern China and analyzed for pH, soil organic matter (SOM), Fe, Mn, and As. The results indicate that SOM accumulated in the upper horizons, while Fe and Mn were reductively leached from the upper horizons and significantly accumulated in the lower argillic horizons. Atmospheric As deposition and As redistribution during the pedogenic process led to the unique vertical distribution of As concentrations in the wetland Argialbolls. Overall, As was leached from upper horizons and then accumulated in the lower argillic horizons. However, continual atmospheric As deposition maintained a slightly elevated As concentration in the top layer. In detail, As concentration in the upper horizons ranged from 1.1 to 5.3 mg kg^−1^, while it ranged from 18.2 to 65.7 mg kg^−1^ in the lower argillic horizons. The high As concentration in the argillic horizons might pose a risk to shallow groundwater in the area.

Arsenic is ubiquitous in the environment and is widely recognized as being among the most toxic of the chemical elements^1^,^2^. Arsenic in soils is derived from both its parent materials and anthropogenic sources such as mining, smelting, the use of As-containing pesticides and animal manures, and irrigation with As-contaminated groundwater^1^,^2^,^3^. Arsenic in soils can enter into the food chain via plant uptake and then pose a potential risk to human health, especially for rice consumers because rice is naturally an As-accumulating plant, a feature attributed to its culture in paddy soils^4^,^5^,^6^.

The vertical distribution of As in soil profiles is investigated for the identification of its transport and anthropogenic influences. Peryea and Creger^7^ examined the vertical distribution of As in the six contaminated orchard soils in the state of Washington, finding that As concentration increased at first and then decreased with depth, with a concentration peak at a depth of approximately 25 cm. In the forest soil profiles (Haplocryod and Cryorthent) of northern Sweden, As accumulated in the O horizon or B horizon^8^. In the soil profiles of Guangdong province of southeastern China, As concentration had an increasing tendency of 10.4, 10.7 to 11.3 mg kg^−1^ from the A-, B- to C-horizons, respectively, showing that the distribution was mainly controlled by parent materials^9^. In Missouri soils having cambic or argillic horizons, As significantly accumulated in the argillic or cambic horizon^6^.

While some studies have investigated the distribution of As concentrations in soil profiles, most of the existing studies dealt with agricultural or forest ecosystems. Much less research has been conducted regarding wetland ecosystems. In addition, to our knowledge, As concentration distributions in Argialbol profiles have not been reported until now. Such are the conditions in the Sanjiang Plain in northeastern Heilongjiang Province of China, which has Argialbol soils and where wetland is a major natural ecosystem.

Therefore, the objective of this study was to investigate the vertical distribution of As concentration in the wetland Argialbolls of the Sanjiang Plain, northeastern China. This information is important to understand pedogenic processes and anthropogenic impacts on As in Argialbolls.

## Results and Discussion

### General properties of soil profiles

**Figure 1:**
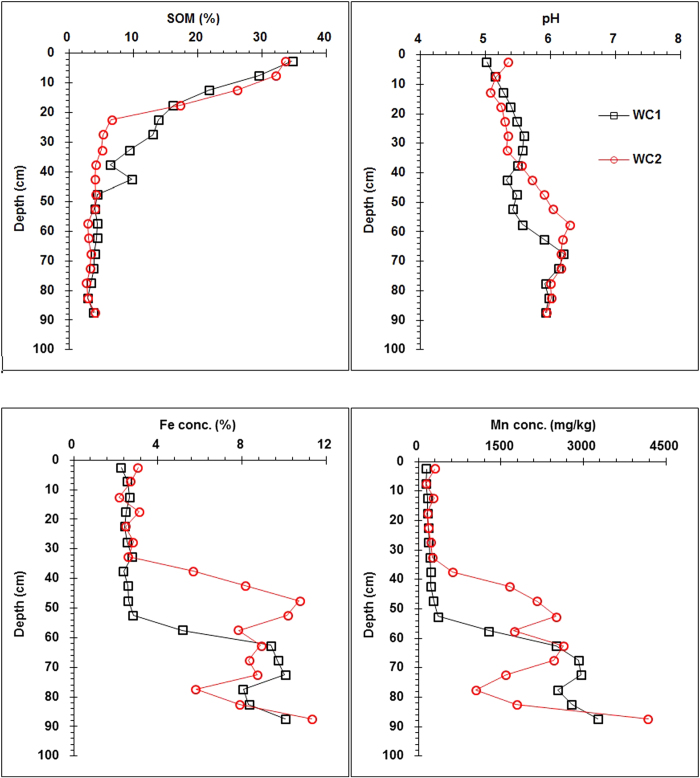


pH, SOM, Fe, and Mn concentrations of soil profiles are shown in Fig. 1. The pH for wetland soil profiles increased from 5-5.2 in the surface horizon to 6.2 in the bottom horizon, indicating that the soil was slightly acidic.

SOM concentration in the wetland soil generally decreased from approximately 34% at 0 to 5 cm depth to approximately 4% at 45 to 50 cm depth (Fig. 1). Below 50 cm depth, SOM concentrations in the soil generally did not change with depth. The vertical distribution of SOM content is similar to that of soil organic carbon in the Sanjiang Plain, as reported by Chi *et al.*^10^.

In contrast to SOM, Fe and Mn were accumulated in the lower horizons of the wetland soil (Fig. 1). In the Fe and Mn oxide-poor upper horizons of the wetland soil (0 to 55 cm depth for core 1 and 0 to 35 cm depth for core 2, where SOM generally accumulated), Fe and Mn concentrations were only approximately 2.2 to 2.8% and 145 to 281 mg kg^−1^, respectively. However, in the Fe and Mn oxide-rich lower horizons of the wetland soil (55 to 90 cm depth for core 1 and 35 to 90 cm depth for core 2), Fe and Mn concentrations were generally 5.2 to 11.3% and 1048 to 3473 mg kg^−1^, respectively. The vertical distributions of Fe, Mn, and SOM concentrations in the wetland soil (Argialbolls) demonstrated that Fe and Mn were reductively leached from the upper horizons and accumulated in the lower horizons. Similar vertical distributions of Fe and Mn concentrations were observed in the Dystric Cambisol profile of Austria, where the Fe and Mn content increased from 2.8% and 160 mg kg^−1^ at 1.5 cm depth to 11.1% and 1596 mg kg^−1^ at 200 cm depth, respectively, due to long-term weathering and leaching^11^.

### As concentration profiles for wetland soil

**Figure 2:**
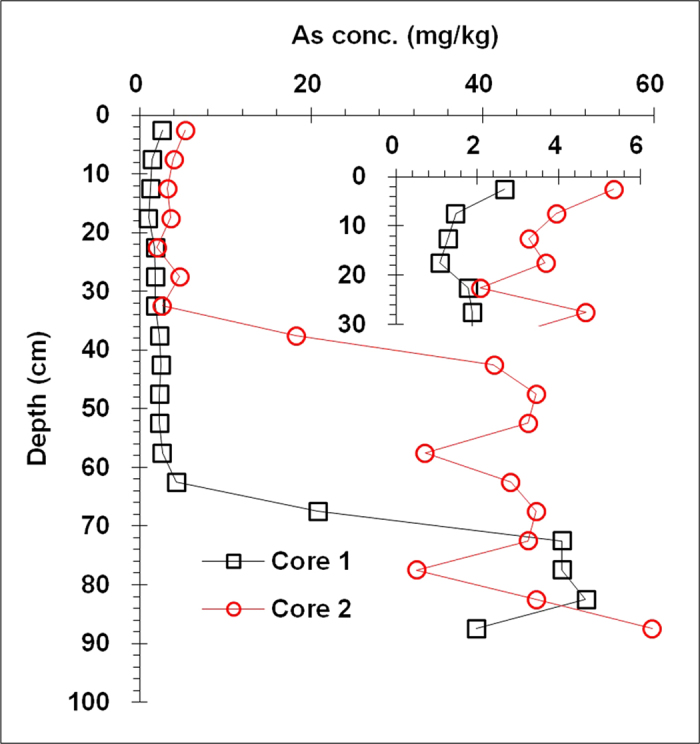


The As concentration profiles for the wetland Argialbolls are shown in Fig. 2. The vertical distribution of As concentrations was generally similar to that of Fe and Mn concentrations.

In the Fe and Mn oxide-poor upper horizons of the wetland soil, As concentrations ranged from 1.1 to 5.3 mg kg^−1^. In the Fe and Mn oxide-rich lower horizons of wetland soil, however, As concentration ranged from 18.2 to 59.8 mg kg^−1^., much higher than the median concentration of As (6 mg kg^−1^) in worldwide soils^12^.

Soil organic matter, clay minerals, and hydrous metal oxides are important solid phase adsorbents for dissolved As and may affect its movement^7^. In addition, pH and competitive ions such as phosphate might also affect As adsorption and mobility^13^.

The vertical distribution of As concentrations in the wetland Argialbolls of the Sanjiang Plain may be caused by following possible mechanisms. First, atmospheric As deposition might lead to slightly elevated As concentration in the surface horizon. Atmospheric deposition is the only input of anthropogenic As at the studied site. China’s total atmospheric As emission from coal combustion has rapidly increased from 635.57 × 10^3^ kg in 1980 to 2205.50 × 10^3^ kg in 2007^14^. The annual total of atmospheric As emissions in 2010 was estimated to be 56.22 × 10^3^ kg from Heilongjiang Province^15^, where the study site is located. The average annual As dissipation rate into the pedosphere over the industrial age is 26.48 × 10^6^ kg a^−1^ 3. In the rural area of China, the average As content in the atmosphere was 47.66 mg kg^−1^, while the dry deposition flux of As was 1.7 mg m^−2^ a^−1^ 16. The average atmospheric dry and wet deposition fluxes of As ranged from 0.41 to 1.93 mg m^−2^ a^−1^ and 0.14 to 4.97 mg m^−2^ a^−1^ in the Chiayi County in Southern Taiwan, respectively^17^. A previous study showed that anthropogenic As from atmospheric deposition led to a greater accumulation of As in the O horizons of Haplocryod and Cryorthent in northern Sweden^8^. Second, low As concentration in the upper horizons may be mainly caused by leaching coupled with production and volatilization of alkylarsines in the anaerobic soil. Arsenite formed in anaerobic soils is more soluble and mobile than arsenate^18^. Arsenic adsorbed to the upper soil horizons can be displaced by phosphate through a mechanism of competitive anion exchange and then can be readily leached into subsoil^19^. Yang *et al.*^20^ observed that phosphorus concentrations in the upper horizons of the wetland soil usually ranged from 1000 to 2600 mg kg^−1^; thus, anion exchange adsorption between arsenate and phosphate might occur in the wetland soil. In addition, arsenic can be lost from soils because of microbially mediated production of volatile alkylasines by bacteria in anaerobic soils^21^. Third, the high As concentration in the low argiallic horizons was caused by the adsorption on Fe and Mn oxides of leached As from upper horizons. Adsorption of As onto Fe oxides exhibits a shoulder like maximum between pH 4 and pH 7^8^. Hence, slightly acidic conditions in the lower argiallic horizons of the wetland soil favors As adsorption and its accumulation. Aide *et al.*^6^ also documented the significant accumulation of As in the argillic horizons in Missouri soils (Alfisols, Ultisols, and Mollisols).

### Correlation between As and mineral matrix elements Fe and Mn

The concentration of As in the wetland soil cores was positively correlated with the concentrations of Mn and Fe very closely (R^2^ values of 0.9241 and 0.9745, respectively) and significantly (*p* < 0.001) (Fig. 3). These correlations further demonstrate the strong affinity between As and Fe and Mn oxides in the wetland Argialbolls of the Sanjiang Plain. Most As was nominally associated with the Fe-Mn oxide and organic fractions in three tea garden soil profiles in the state of Assam, India^22^.

The wetland Argialbol profiles in the Sanjiang Plain of northeastern China are characterized by SOM accumulation in the upper horizons, Fe and Mn leaching from upper horizons and subsequently their accumulation in lower horizons, and a slightly acidic pH. The vertical distributions of As concentration in the wetland Argialbolls are controlled by the distribution of adsorbents such as SOM and Fe and Mn oxides, atmospheric As deposition, and biogeochemical processes such redox and desorption/sorption in the wetland soils. These comprehensive factors led to the unique character of the vertical distributions of As concentration in the wetland Argialbolls of the Sanjiang Plain. On one hand, As concentrations were much higher in the lower argillic horizons than in the upper SOM-rich horizons. On the other hand, atmospheric As deposition led to a slight accumulation of As in the topsoil.

## Materials and Methods

### Description of study area

The Sanjiang Plain, an area of low relief within the Heilongjiang Province of China, is located in the temperate climate zone, which is characterized by a mean annual temperature of 3 °C and an annual precipitation ranging from 500 to 600 mm. Water and soil are completely frozen from late October to early April, and the highest temperatures are found in July. Three rivers, the Amur, Songhua, and Ussuri, provide the major waterway system and alluvial deposits in this area. Since the onset of the Quaternary period, the landmass in the Sanjiang Plain has gradually evolved into the flat landscape of today. Underlain by a continuous clay layer, the Sanjiang Plain has a slope grade of less than 1:10,000, which is favorable for wetland formation^23^.

The wetland soil in the Sanjiang Plain is generally classified as a Histosol (Argialbol)^24^ and is generally characterized by the accumulation of organic matter in the A horizon and migration of clay minerals from the E horizon (albic horizon) and their accumulation in the Bt horizon (argillic horizon). The parent materials are quaternary alluvial sediments. The soil formation processes are strongly affected by frequent wetting-drying cycles leading to the alternation of oxidation-reduction processes^25^.

### Soil core collection and analysis

Wetland soil cores were collected with single gouge augers (Eijkelkamp, Netherland) from the natural wetland in late May 2010. The wetland, where two soil cores (134.0884E and 47.4035N) were taken, is generally not influenced by local agricultural activity. The distance between these two soil cores is approximately 500 m. The cores were 90 cm long, and they were sliced into 5-cm slices.

The soil samples were transferred to acid-washed, dark-colored polyethylene bags and transported to the laboratory where they were freeze-dried, slightly crushed, passed through a 2-mm sieve and stored in glass bottles until analysis.

The pH of each soil sample was analyzed in a 1:10 solid/liquid ratio suspension using a combination pH electrode. The organic matter concentration was measured by weight loss on ignition at 400 °C^26^. Portions of the soil samples were digested with HNO_3_–HF–HClO_4_ 27. The Fe and Mn in the extracts were measured with Inductively Coupled Plasma Atomic Emission Spectrometry (ICP-AES) (IRIS Intrepid II, Thermo Electron).

Another aliquot of soil was digested with aqua regia (1% KMnO_4_) and 1% oxalic acid. The As in the supernatant was determined by hydride generation atomic fluorescence spectrometry (HGAFS).

## Additional Information

**How to cite this article**: Lin, C. *et al.* Arsenic profile distribution of the wetland argialbolls in the Sanjiang plain of northeastern China. *Sci. Rep.* **5**, 10766; doi: 10.1038/srep10766 (2015).

## Figures and Tables

**Figure 3:**